# Al/Si Nanopillars as Very Sensitive SERS Substrates

**DOI:** 10.3390/ma11091534

**Published:** 2018-08-26

**Authors:** Giovanni Magno, Benoit Bélier, Grégory Barbillon

**Affiliations:** 1 Centre de Nanosciences et de Nanotechnologies, CNRS, University Paris Sud, Université Paris-Saclay, C2N-Orsay, CEDEX, 91405 Orsay, France; giovanni.magno@c2n.upsaclay.fr (G.M.); benoit.belier@c2n.upsaclay.fr (B.B.); 2 EPF-Ecole d’Ingénieurs, 3 Bis Rue Lakanal, 92330 Sceaux, France

**Keywords:** SERS, sensors, aluminum, silicon

## Abstract

In this paper, we present a fast fabrication of Al/Si nanopillars for an ultrasensitive SERS detection of chemical molecules. The fabrication process is only composed of two steps: use of a native oxide layer as a physical etch mask followed by evaporation of an aluminum layer. A random arrangement of well-defined Al/Si nanopillars is obtained on a large-area wafer of Si. A good uniformity of SERS signal is achieved on the whole wafer. Finally, we investigated experimentally the sensitivity of these Al/Si nanopillars for SERS sensing, and analytical enhancement factors in the range of 1.5 × 10${}^{7}$ − 2.5 × 10${}^{7}$ were found for the detection of thiophenol molecules. Additionally, 3D FDTD simulations were used to better understand optical properties of Al/Si nanopillars as well as the Raman enhancement.

## 1. Introduction

During this last decade, Surface Enhanced Raman Scattering (SERS) is mainly employed as a powerful technique for detection of biological/chemical molecules. The fabrication of SERS substrates having high enhancement factors (EF) is the key point for the improvement of this biological/chemical sensing. Several groups investigated a great number of novel SERS substrates, which demonstrated a large Raman enhancement, such as colloidal metallic nanoparticles [1,2,3] and metallic nanostructures on different surfaces fabricated by various lithographic techniques [4,5,6,7,8,9,10,11]. Indeed, this large Raman enhancement is mainly due to the presence of hotspots in these different SERS substrates. The mechanism of this enhancement due to the hotspots is well-described in References [12,13], and the development of this type of SERS substrates with high densities of hotspots is demonstrated in References [8,9,14,15,16,17]. However, certain fabrication techniques cited previously are technologically demanding in terms of time and expensive for a mass production destined to industrial applications. Besides, Nanoimprint Lithography (NIL) [18,19,20] and Nanosphere Lithography (NSL) [21,22,23] allows fabricating these SERS substrates with a lower cost. Nevertheless, they can be plagued by poor definition of nanostructures obtained on large surfaces, which are required for practical/industrial applications. Another way for obtaining higher EFs is to use silicon nanowires (SiNW) coupled to metallic nanoparticles. This type of nanostructures allows obtaining a better detection limit [16,24,25]. Moreover, disordered SiNWs can be fabricated by large-surface techniques. Although all these SERS substrates have great potential for a very sensitive detection of chemical or biological molecules, most of the applications are hampered by the non-uniformity of the SERS signals. Several groups have already addressed this non-uniformity issue of the SERS signal, and they have demonstrated a good uniformity of the latter [26,27].

In this paper, the aim is to present a simple and fast process to produce very sensitive SERS substrates composed of Al/Si nanopillars at the large-area wafer-scale, which will have a good uniformity of the SERS signal. In our case, aluminum was chosen as a plasmonic material for its attractive properties including low cost, high natural abundance, compatibility with CMOS technology and optoelectronic devices, and plasmonic resonances in the spectral domains of UV and visible [28,29]. Moreover, aluminum plasmonics can be applied to a wide range of applications such as SERS in ultraviolet [30] and visible domains [31,32,33], SEIRA (Surface-Enhanced Infrared Absorption Spectroscopy) [31,34], photocatalysis [35], and metal-enhanced fluorescence [36]. Although several groups have already worked on Al plasmonics nanostructures for SERS sensing [30,31,34], there was little consideration on the synergy between silicon and aluminum in order to improve the performance of SERS sensors. Here, the ability of Al/Si nanopillars to be very sensitive SERS sensors is investigated and evaluated using thiophenol solutions. To further deepen the comprehension of the SERS signal enhancement, 3D FDTD simulations are made.

## 2. Experimental Details

### 2.1. Two-Step Fabrication of Al/Si Nanopillars

**Figure 1:**
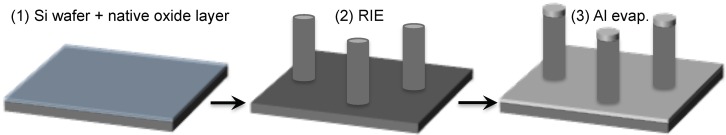
Principle scheme of the Al/Si nanopillar fabrication. The metal evaporation step is made under normal incidence.

The fabrication process of large area of Al/Si nanopillars (NPs) is composed of two steps (see Figure 1): (i) etching through the mask obtained by the native oxide layer; and (ii) depositing of titanium and aluminum layers under vacuum by Electron Beam Evaporation (EBE). In this fabrication process, no pre-patterning of the Si surface is required. Indeed, we only use the native oxide layer of Si wafer as a physical etch mask. Then, an anisotropic RIE (Reactive Ion Etching) process consisting in sixty cycles of passivation and etching steps is realized on the Si wafer through the native oxide layer by using ICP-SPTS (Inductively Coupled Plasma-Sumitomo Precision Products Process Technology Systems) equipment. Gases involved in this protocol are SF_6_ (300 sccm), C_4_F_8_ (180 sccm) and O${}_{2}$ (200 sccm). This anisotropic RIE process is a switched process in which fluorine from SF${}_{6}$ etches the Si while C${}_{4}$F${}_{8}$ passivates the surface, and it starts with a cycle of passivation. During this first and short passivation cycle, only certain nanoscale zones of the native oxide layer are randomly passivated, which will then serve as etch mask and thus produce Si nanopillars at the end of these zones. The organization of the obtained nanopillars is completely random. The pressure and power used in this process are 20 mTorr and 25 W, respectively. By modifying the process parameters such as cycle times, number of cycles, platen and coil power, and substrate temperature, the size distribution, depth and density can be controlled. To finish, a 2 nm titanium layer used as adhesion layer, and an aluminum layer of 50 nm are deposited by EBE under normal incidence. The evaporation rate used in this process are 0.05 nm/s and 0.3 nm/s for Ti and Al layers, respectively.

### 2.2. Thiophenol Deposition on Al/Si Nanopillars

For our SERS investigations, thiophenol molecules were used to test the sensitivity of these Al/Si nanopillars because they are excellent model molecules. The deposition protocol is as follows: (i) preparation of a 1 μm solution of thiophenol in ethanol; (ii) dipping the SERS sample in the solution for 3 h; and (iii) the SERS sample was allowed to nitrogen dry in a specific box. For our reference experiment, the deposition protocol is: (i) preparation of a 1 M solution of thiophenol in ethanol; (ii) dipping the reference sample (Si substrate with nanopillars without metal) in the solution for 3 h; and (iii) the reference sample was allowed to nitrogen dry in a specific box.

### 2.3. Raman Characterization

For all the Raman measurements, we employed a Labram spectrophotometer from Horiba Scientific, which has a spectral resolution of 1 cm${}^{-1}$. The excitation wavelength ($\lambda_{exc}$ = 633 nm) and an acquisition time of 10 s were used for all the SERS and Raman (reference) experiments. For these characterizations, the laser was focused on the substrate using a microscope objective (100×, N.A. = 0.9). The Raman signal from the SERS substrates (or reference experiment) was collected by the same objective in a backscattering configuration, and the used laser power was 1 mW. The average of SERS intensities and relative standard deviations (RSD) were calculated on the basis of 25 SERS spectra.

### 2.4. FDTD Simulations of SERS Substrates

**Figure 2:**
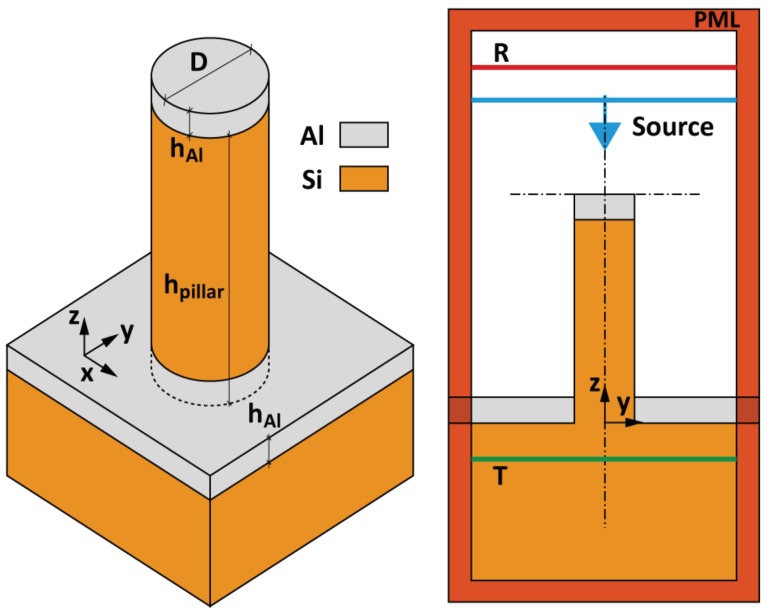
Nanopillar morphology used for the FDTD simulations, the diameter (D) is 150 nm, the height (h*_pillar_*) is 1450 nm, and the Al layer thickness (h*_Al_*) is 50 nm on the top of nanopillar and on Si substrate. On the right, the broadband plane wave source and monitors (R and T) for calculating the extinction spectrum are displayed.

**Figure 3:**
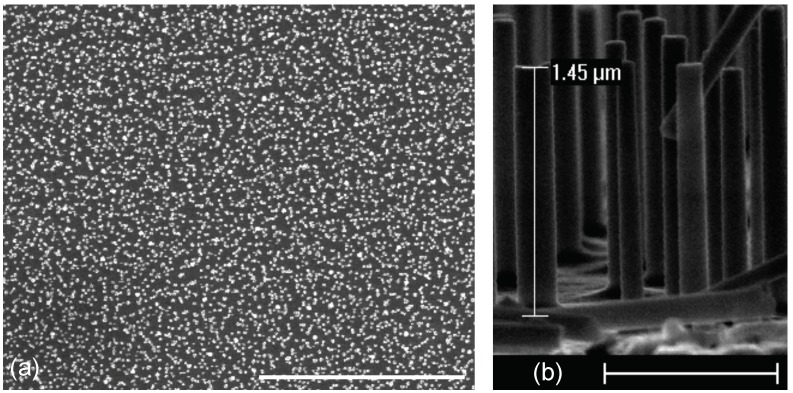
SEM images of Al/Si nanopillars obtained with our fabrication technique: (**a**) on a large zone (scale bar = 20 μm); and (**b**) cross-section view of the nanopillars (scale bar = 1 μm).

To calculate the extinction spectrum of the SERS substrates, 3D Finite-Difference Time-Domain (FDTD) method was used. For these FDTD simulations, we considered an isolated Al/Si nanopillar, which corresponds to the experimental case for Al/Si nanopillars (see Figure 2 and Figure 3). The nanopillar diameter (D) is 150 nm, its height (h*_pillar_*) is 1450 nm, and the Al layer thickness (h*_Al_*) is 50 nm on the top of nanopillar and on Si substrate. The top corners of the Al/Si nanopillar are not rounded. Both materials used for this study have been modelled by fitting the real and imaginary parts of the permittivities reported in the reference [37]. The nanopillar on substrate, centred in a computational cell of 3 × 3 × 5 μm^3^, is surrounded by Perfectly Matched Layers (PML) in order to absorb radiation leaving the calculation region. For providing an excellent resolution of the fields, a uniform mesh of 2 × 2 × 2 nm${}^{3}$ was used for discretising the computational cell. Finally, the extinction spectrum has been calculated by exciting the structure with a broadband plane wave source (spectral range from 400 nm to 800 nm) impinging from above the pillar and by collecting the reflected (R) and transmitted (T) powers. This simulated extinction spectrum does not consider the thiophenol layer. Thus, these simulations shed light on the optical properties of Al/Si nanopillars.

## 3. Results and Discussion

Firstly, Al/Si nanopillars were fabricated with the process in Section 2.1. Figure 3 displays SEM images of these Al/Si nanopillars. The diameter and the height of the Al/Si NPs were determined to be 150 ± 40 nm, and 1450 ± 50 nm, respectively. The homogeneity of Al/Si nanopillars is correct in terms of dimensions.

**Figure 4:**
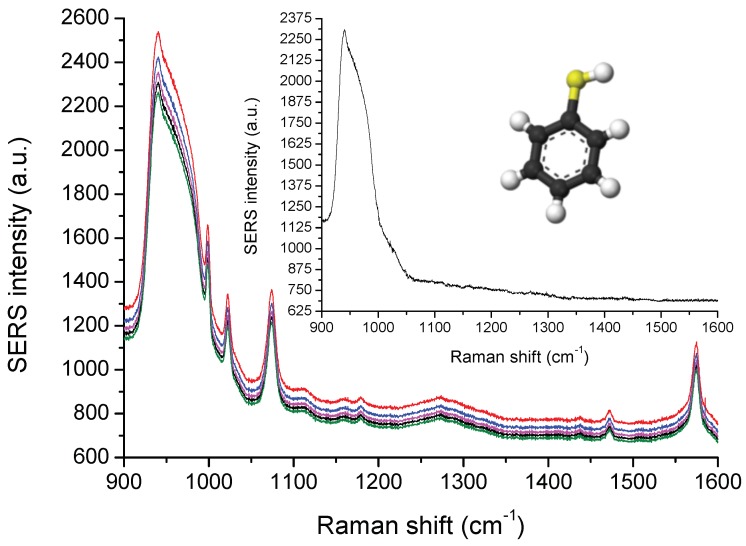
Five SERS spectra of thiophenol molecules recorded randomly on the whole substrate composed of Al/Si nanopillars. The inset depicts the Raman spectrum of thiophenol obtained with only Si nanopillars (without metal). Moreover, the molecular scheme of the thiophenol molecule is also displayed.

Next, thiophenol molecules (see molecular scheme in Figure 4) were deposited on Al/Si NPs directly after their fabrication with the protocol in Section 2.2, and then characterized directly by Raman measurements. Figure 4 reveals the SERS spectra of thiophenol on Al/Si nanopillars recorded at the excitation wavelength of 633 nm. On all SERS spectra, we observed Raman shifts, which are characteristic of thiophenol molecules [38,39,40] as those at 1000 cm${}^{-1}$ corresponding to the C-C stretching mode (named: ν(CC), see References [40,41,42]); at 1025 cm${}^{-1}$ corresponding to the combination of the following modes: C-C stretching and C-H in-plane bending (named: ν(CC) and δ(CH), respectively, see References [40,41,42]); at 1075 cm${}^{-1}$ corresponding to the combination of the following modes: C-C stretching, C-H in-plane bending and C-S stretching (named: ν(CC), δ(CH) and ν(CS), respectively, see References [40,41,42]); and at 1575 cm${}^{-1}$ corresponding to the C-C stretching (named: ν(CC), see References [40,41,42]). Besides, some multi-phonon peaks of Si in the range of 900–980 cm${}^{-1}$ are observed [43,44]. In the inset of Figure 4, a reference Raman spectrum of thiophenol obtained with only Si nanopillars (without metal) is displayed. No significant Raman shift studied here is visible, because they are very weak.

To evaluate the sensitivity of Al/Si nanopillars, the analytical enhancement factor (AEF) is calculated for the 4 characteristic Raman peaks of the thiophenol molecules previously cited. *AEF* is given by the following formula:

(1) $$\begin{array}{c} AEF=\frac{I_{SERS}}{I_{Raman}}\times \frac{C_{Raman}}{C_{SERS}} \end{array}$$

where $I_{SERS}$, $I_{Raman}$ represent the SERS and Raman intensities, respectively (see Table 1). $C_{SERS}$ (1 μm), $C_{Raman}$ (1 M) are the concentrations of thiophenol for SERS and reference Raman experiments, respectively.

From the results in Table 1, the largest AEF value, which was found for the Al/Si nanopillars, is 2.4 × 10${}^{7}$ the Raman shift of 1575 cm${}^{-1}$. In addition, some groups have obtained excellent AEFs of ∼10${}^{6}$ with Ag nanoparticles on Si/ZnO nanotrees [45] and around 2 × 10${}^{6}$ with Au nanostructured electrodes [46]. Furthermore, other groups have demonstrated good EF with similar SERS substrates such as Ag nanoparticles on Si nanowires (for [17]: EF∼4 × 10${}^{6}$; for [16]: EF = 10${}^{7}$− 2.3 × 10${}^{8}$; and for [24]: EF = 10${}^{8}$− 10${}^{10}$), and Si nanopillars covered on the nanopillar top by Ag lumps (EF ∼5 × 10${}^{6}$) [25]. By comparison, our Al/Si nanopillars are faster to fabricate and a better sensitivity is achieved for all the Raman peaks studied here (1.5 × 10${}^{7}$ < AEF < 2.5 × 10${}^{7}$) except for Ag nanoparticles on Si nanowires of References [16,24] concerning to the sensitivity. Besides, the relative standard deviation (RSD) is calculated for all the four peaks of our investigation in order to quantify the uniformity. To do that, 25 SERS spectra of thiophenol molecules were recorded from several randomly chosen zones on the whole wafer under same experimental conditions. In Table 1, a good uniformity (RSD <7%) of SERS signal is obtained for each Raman peak on the large-area wafer of the Al/Si nanopillars.

**Figure 5:**
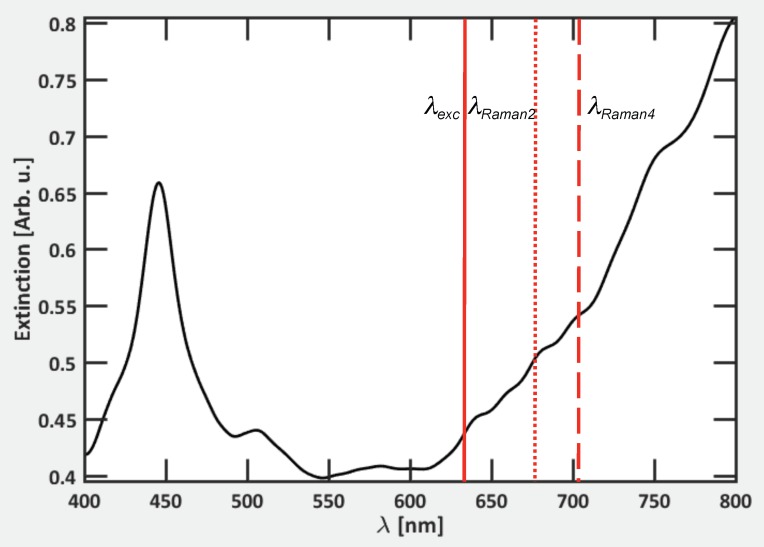
Calculated extinction spectrum of Al/Si nanopillars. $\lambda_{exc}$ corresponds to the excitation wavelength ($\lambda_{exc}$ = 633 nm, continuous red line). $\lambda_{Raman2}$ and $\lambda_{Raman4}$ correspond to the Raman scattering wavelengths for the Raman shifts of 1025 cm${}^{-1}$ and 1575 cm${}^{-1}$ ($\lambda_{Raman2}$ = 677 nm, dotted red line, and $\lambda_{Raman4}$ = 703 nm, dashed red line), respectively. For the sake of readability, only $\lambda_{Raman2}$ and $\lambda_{Raman4}$ are displayed, since $\lambda_{Raman1}$ and $\lambda_{Raman3}$ are very close to $\lambda_{Raman2}$.

To understand these experimental results, we calculated the extinction spectrum of the SERS substrate (see Figure 5). From this, we easily observe the positions of different resonances observed for these Al/Si nanopillars compared to the positions of the excitation wavelength and Raman wavelengths associated to the Raman shifts measured experimentally. Moreover, $\lambda_{Raman}$ is the Raman scattering wavelength corresponding the studied Raman shift, which is determined with the following formula:

(2) $$\begin{array}{c} \Delta \omega =10^{7}\left(\frac{1}{\lambda_{exc}}-\frac{1}{\lambda_{Raman}}\right) \end{array}$$

where Δω is the studied Raman shift (in cm${}^{-1}$), $\lambda_{exc}$ is the excitation wavelength used in the experiments (in nm), and $\lambda_{Raman}$ is the Raman scattering wavelength to be determined (in nm, see Table 1).

Finally, we can qualitatively analyze the SERS enhancement, which can be obtained by using the E${}^{4}$ model, assuming that enhancement factor is proportional to the extinction intensities (Q${}_{e}$) at $\lambda_{exc}$ and $\lambda_{Raman}$, i.e., EF ∼ Q${}_{e}$($\lambda_{exc}$) × Q${}_{e}$($\lambda_{Raman}$) [47]. In Figure 5 and Table 1, we observe that EF${}_{4}$ is the highest value, and the EF values increased when $\lambda_{Raman}$ also increased, i.e., Q${}_{e}$($\lambda_{Raman}$) increased with $\lambda_{Raman}$. The different EF values correspond to enhancement factors for the couples ($\lambda_{exc}$, $\lambda_{Raman1}$), ($\lambda_{exc}$, $\lambda_{Raman2}$), ($\lambda_{exc}$, $\lambda_{Raman3}$) and ($\lambda_{exc}$, $\lambda_{Raman4}$), respectively. These FDTD results suggest that the AEF values observed experimentally (see Table 1) have the same behavior as the EF values obtained with the E${}^{4}$ model.

## 4. Conclusions

In this paper, we demonstrate the fast fabrication of very sensitive SERS substrates composed of Al/Si nanopillars for chemical detection. The key point of this fabrication process is the use of a native oxide layer as a physical etch mask. This fabrication allowed obtaining well-defined nanopillars at the large-area wafer-scale. The sensitivity of these Al/Si nanopillars was investigated and compared to the results obtained for gold nanostructured electrodes [46], Ag nanoparticles on Si/ZnO nanotrees [45], Ag nanoparticles on Si nanowires [16,17,24], and Si nanopillars covered on the nanopillar top by Ag lumps [25]. The AEF values achieved with our Al/Si nanopillars (1.5 × 10${}^{7}$ < AEF < 2.5 × 10${}^{7}$) is better than the SERS substrates cited previously, except for Ag nanoparticles on Si nanowires of References [16,24]. Moreover, an excellent uniformity of SERS signal (RSD <7%) was achieved on the whole wafer, which is a key point for industrial applications. Thus, such Al/Si nanopillars could be integrated on a lab-on-chip for label-free chemical/biological detection processes. 

## Figures and Tables

**Table 1:** For the excitation wavelength of 633 nm and four Raman peaks (RS) studied here, $\lambda_{Raman}$ associated to RS, the intensities $I_{Raman}$ and $I_{SERS}$, RSDs associated to $I_{SERS}$ values, analytical enhancement factors (AEF) and EF values (in arbitrary unit) obtained with the $E^{4}$ model are presented.

Name	RS (cm${}^{-1}$)	$\lambda_{Raman}$ (nm)	$I_{Raman}$	$I_{SRRS}$	RSD (%)	AEF	EF (a.u.)
1	1000	676	16	271	6.6	1.7 × 10${}^{7}$	0.220
2	1025	677	11	209	4.8	1.9 × 10${}^{7}$	0.222
3	1075	679	20	409	4.4	2.1 × 10${}^{7}$	0.224
4	1575	703	14	334	3.6	2.4 × 10${}^{7}$	0.238
